# Independent predictive value of the monocyte-to-lymphocyte ratio for severe cardiopulmonary symptoms in high altitude polycythemia

**DOI:** 10.3389/fphys.2026.1986276

**Published:** 2026-10-20

**Authors:** Lu Dai, Wentao Wu, Tengyun Chen, Wenjin Sun, Qi Zhang, Qingqing Su, Yuenan Ni, Xuan Zhang

**Affiliations:** 1 Department of Pulmonary and Critical Care Medicine, West China Hospital, Sichuan University, Chengdu, Sichuan, China; 2 High Altitude Medicine Key Laboratory of Sichuan Province, West China Hospital of Sichuan University, Chengdu, Sichuan, China; 3 Department of High Altitude Medicine, Center for High Altitude Medicine, West China Hospital of Sichuan University, Chengdu, Sichuan, China; 4 Department of Respiratory Care, West China School of Medicine and West China Hospital, Sichuan University, Chengdu, Sichuan, China; 5 Department of Neurosurgery, West China Hospital, Sichuan University, Chengdu, China

**Keywords:** chronic mountain sickness, high altitude polycythemia, immunophenotyping, monocyte-to-lymphocyte ratio, risk stratification

## Abstract

**Background:**

High altitude polycythemia (HAPC) is traditionally viewed as a homogeneous condition driven by absolute hypoxemia, while clinical symptom severity fails to correlate with hemoglobin levels. This study aimed to identify distinct clinical phenotypes in HAPC and evaluate novel predictive biomarkers.

**Methods:**

A total of 198 patients with HAPC were analyzed. Unsupervised K-means clustering was performed based on standardized absolute counts of five peripheral leukocyte subsets. A pre-specified multivariable Firth penalized logistic regression and receiver operating characteristic (ROC) analysis were utilized to evaluate the predictive capacity of the monocyte-to-lymphocyte ratio (MLR).

**Results:**

Clustering identified four distinct immunophenotypes. Baseline demographics, absolute residential altitude, and hemoglobin levels were statistically comparable across all groups. Interestingly, Cluster 2 emerged as a highly symptomatic cardiopulmonary phenotype, with quiescent systemic inflammatory markers and the lowest MLR but the highest incidence of breathlessness and palpitations (32.3%). Multivariable analysis demonstrated that patients in the lowest MLR quartile exhibited a significantly elevated risk of belonging to the Cluster 2 phenotype (OR: 33.764, 95% CI: 9.615-180.993, P<0.001) compared to those in the highest quartile. Furthermore, MLR demonstrated favorable diagnostic discrimination for identifying this specific phenotype, with an optimism-corrected area under the curve of 0.810.

**Conclusion:**

HAPC exhibits substantial pathophysiological heterogeneity independent of erythrocytosis severity. The MLR serves as a potential, independent, and accessible surrogate biomarker for the early identification of patients at risk for severe cardiopulmonary symptoms, facilitating precision triage in high-altitude medicine.

## 1 Introduction

Long-term exposure to high-altitude hypobaric hypoxia triggers various physiological adaptations, with the most prominent being compensatory erythrocytosis (León-Velarde et al., 2005; Villafuerte et al., 2022). However, chronic maladaptation to this extreme environment frequently leads to high altitude polycythemia (HAPC). As a core manifestation of chronic mountain sickness (CMS), HAPC is characterized by excessive red blood cell proliferation and severe hyperviscosity, which predispose patients to multi-organ dysfunction and a remarkably poor prognosis (Villafuerte and Corante, 2016; Penaloza and Arias-Stella, 2007).

As the disease progresses under sustained hypoxic exposure, patients with HAPC often experience a progressive exacerbation of clinical symptoms, reflected by increasing CMS scores, which further amplifies the risk of adverse cardiovascular and neurological events (Champigneulle et al., 2022). Historically, the absolute magnitude of erythrocytosis was presumed to be the primary determinant of disease severity. However, accumulating clinical evidence has revealed that extreme polycythemia does not reliably serve as a clinical surrogate for CMS severity (Hancco et al., 2020; Oberholzer et al., 2020). Multiple studies have demonstrated that absolute hemoglobin (Hb) levels often fail to correlate with subjective CMS clinical scores or the precise extent of end-organ damage (Gonzales et al., 2011a, b, 2013). Consequently, accurately evaluating the true severity of HAPC and effectively predicting its risk of progressing to severe CMS complications remain fundamentally unresolved.

This critical discrepancy strongly suggests that HAPC is not a singular, uniform condition driven solely by blood viscosity or a simple dose-response to hypoxemia. Instead, it is a complex and heterogeneous syndrome (Tang et al., 2024; Zhou et al., 2025). Beyond erythropoiesis, chronic hypoxia actively drives systemic inflammatory cascades and immune remodeling (Pham et al., 2021; Zhong et al., 2026). Specifically, hypoxia-inducible factors (HIFs) drive the mobilization and activation of circulating monocytes, promoting their migration into target organs to perpetuate localized inflammation (Lin and Simon, 2016). Concurrently, chronic hypoxic stress frequently induces T-lymphocyte apoptosis, leading to a relative depletion of the circulating lymphocyte pool (Sun et al., 2010). This imbalance between leukocyte lineages, often reflected by the monocyte-to-lymphocyte ratio (MLR), implies that patients facing identical environmental altitude and exhibiting matched Hb levels may traverse distinct pathophysiological trajectories, manifesting divergent clinical symptom profiles.

Given this intrinsic heterogeneity, traditional single-dimensional assessments are inadequate. Unbiased stratification strategies are required to decode the true clinical spectrum of this disease. Therefore, the present study employed an unsupervised machine learning approach to systematically evaluate patients with HAPC. By deconstructing this heterogeneous population, we aimed to identify distinct clinical and biochemical phenotypes and, crucially, to discover novel, accessible predictive biomarkers capable of early risk stratification for severe altitude-related complications.

## 2 Methods

### 2.1 Participants

This cross-sectional study enrolled all adult HAPC patients admitted to West China hospital from September 2014 to September 2025. Strict inclusion required: (1) erythrocytosis defined as [Hb] ≧ 21 g/dL for males and ≧ 19 g/dL for females; (2) permanent residency in high-altitude regions defined as > 2500 m; (3) first-time hospitalization. The exclusion criteria were as follows: (1) diagnosis of polycythemia vera; (2) incomplete data of blood cell counts, especially the differential count; (3)with acute infections or established thrombotic diseases; (4) comorbid respiratory diseases, including chronic obstructive pulmonary disease, asthma, emphysema, chronic bronchitis, bronchiectasis, interstitial lung disease, lung cancer, history of pulmonary surgery and so forth; (5) comorbid cardiovascular disease, including coronary artery disease, myocardial infarction, valvular heart disease, dilated cardiomyopathy, alcoholic/hypertensive cardiomyopathy and so forth; (6) age < 18 years old; and (7) incomplete medical records or inability to comply with physical examinations. The study was approved by the Ethics Committee of West China Hospital, Sichuan University (No. 2024-1930) and complied with the Declaration of Helsinki. A waiver of written informed consent was obtained since all data were retrospectively extracted from electronic medical records (EMRs).

### 2.2 Data collection

Demographic data of each participant were recorded upon admission, including age, sex, and body mass index (BMI). Smoking status, drinking consumptions, and medical conditions were collected. Complete blood count, especially the differential count, C-reactive protein (CRP), D-dimer, and lactate dehydrogenase (LDH) were included. Specifically, the absolute counts of peripheral leukocyte subsets and Hb concentrations were quantitatively measured utilizing a standard automated hematology analyzer at the hospital’s central laboratory upon admission. The neutrophil-to-lymphocyte ratio (NLR) and monocyte-to-lymphocyte ratio (MLR) were subsequently calculated. For altitude calculation and rationale, we used ArcGIS to mosaic and clip Chinese county-level administrative regions for calculating average elevation (https://www.earthdata.nasa.gov/). The traditional medical definition of high altitude is above 2500 m.

### 2.3 Calculation of CMS symptom clinical scores

We extracted the clinical symptoms of HAPC patients from EMRs, including: (1) breathlessness and/or palpitations; (2) sleep disturbance; (3) paresthesia; (4) headache; and (5) tinnitus. Negative responses were assigned to a value of 0, while positive responses were assigned to values of 1 to 3 to indicate mild, moderate, or severe symptoms respectively. Given the retrospective nature of this study, the standardized grading of objective physical signs, specifically dilatation of veins and cyanosis, was inconsistently documented in the EMRs. Therefore, these two objective items were excluded, and our assessment focused exclusively on the subjective symptom burden.

According to the consensus (León-Velarde et al., 2005), 3 points were added if [Hb] levels were ≧ 21 g/dL in males and ≧ 19 g/dL in females designed in the Qinghai CMS Score. Since all enrolled participants were diagnosed with HAPC and inherently met this Hb threshold, they uniformly carried a baseline score of 3. To independently evaluate symptom severity without the confounding weight of the baseline Hb score, we utilized this modified 5-item subjective symptom score. A total score of ≧ 3 on this 5-item scale was utilized to define a highly symptomatic state.

### 2.4 Unsupervised cluster analysis

We employed the absolute counts of the five white blood cell differentials in the cluster analysis, including the neutrophilic segmented granulocyte, monocyte, lymphocyte, eosinophilic granulocyte, and basophilic granulocyte. To eliminate demographic confounding, we employed the K-means algorithm with Euclidean distance, exclusively utilizing standardized (z-score transformed) absolute counts of the five peripheral leukocyte subsets as input features. The optimal cluster number (k=4) was determined by systematically evaluating k values from 1 to 10. We synthesized statistical evaluation metrics, specifically minimizing the total within-cluster sum of squares (WSS) via the elbow method and maximizing the average silhouette width, alongside clinical interpretability. Furthermore, we utilized hierarchical clustering as an alternative algorithmic approach to cross-validate the structural integrity of the identified groups. To quantify the reproducibility of our assignments, we performed bootstrap resampling with 100 iterations and calculated the Jaccard index for each cluster. Regarding feature selection, the standardized scaling transformations were strictly applied to all absolute counts prior to algorithm execution. This critical step was designed to eliminate scale disparities and mitigate potential confounding effects arising from hemoconcentration, plasma volume variations, and potential batch effects or measurement drifts over the long study period. To ensure algorithmic stability and avoid local optima, the K-means clustering incorporated 25 random initializations. High-dimensional cluster separation was subsequently visualized using Principal Component Analysis (PCA) (**Figure 1**).

**Figure 1:**
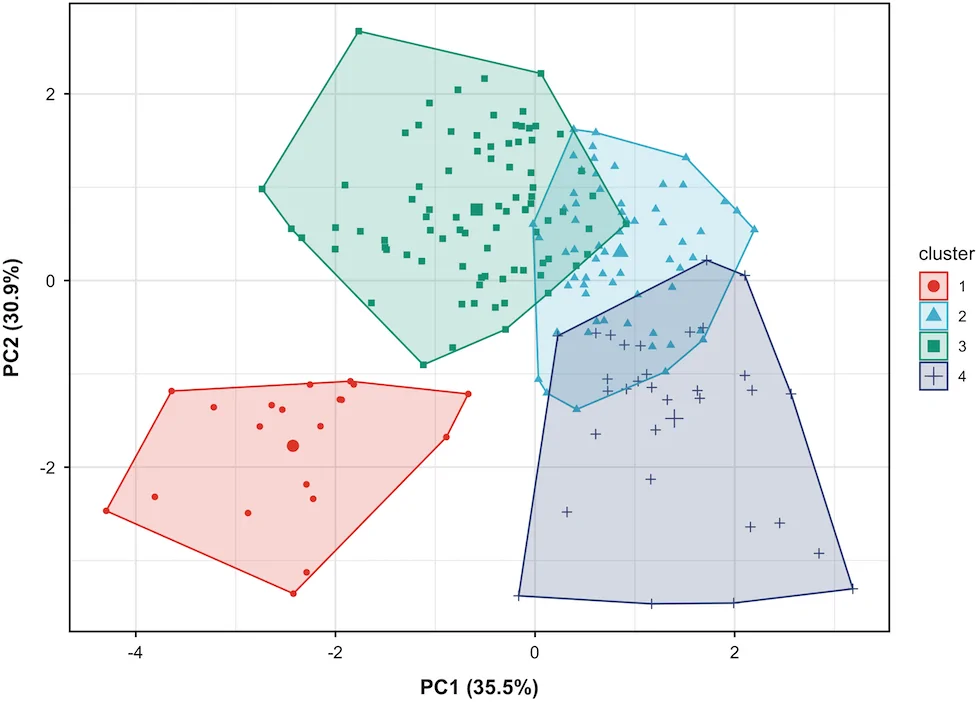
PCA visualization of the four distinct immunophenotypes in HAPC patients. The proportions of variance explained by the PC1 and PC2 are explicitly stated on the respective axes. PCA, Principal Component Analysis; HAPC, high altitude polycythemia; PC, principal components.

### 2.5 Statistical analysis

Categorical data are presented as numbers (percentage). Continuous variables are shown as mean ± standard deviation or median (interquartile range, 25–75%) depending on whether the data were normally distributed or not. Normally distributed data were analyzed using analysis of variance (ANOVA) followed by Tukey HSD *post-hoc* tests. Non-normally distributed data were assessed using the Kruskal-Wallis H test. Spearman rank correlation networks were constructed to evaluate global associations among symptoms, biomarkers, and altitude. Given a missing data rate of 15.38% for the primary leukocyte differentials, we employed a standard complete-case analysis approach. To evaluate the independent predictive capacity of MLR, we employed a pre-specific multivariable Firth penalized logistic regression model to mitigate small sample bias. Covariates including age, BMI, residential altitude, baseline hemoglobin, smoking status, and drinking status were selected *a priori* based on clinical knowledge. For enhanced interpretability, the continuous MLR variable was transformed into quartiles. Model calibration was assessed via the Hosmer-Lemeshow goodness-of-fit test. Diagnostic discrimination was evaluated using receiver operating characteristic (ROC) curves and internally validated utilizing 1000 bootstrap resamples to calculate the optimism-corrected area under the curve (AUC). R (version 4.6.0, R Foundation for Statistical Computing, Vienna, Austria. [https://www.R-project.org/]) was used for data analysis. A two-sided *P*-value <0.05 was considered statistical significance.

## 3 Results

### 3.1 Baseline characteristics and cluster establishment

Based on the standardized absolute counts of the five peripheral leukocyte subsets (neutrophils, lymphocytes, monocytes, eosinophils, and basophils), unsupervised K-means clustering stratified the 198 HAPC patients into four distinct immunophenotypic clusters. The application of the elbow method and silhouette analysis consistently supported four as the optimal number of clusters, as detailed in **Supplementary Figure S1**. Stability analysis via bootstrap resampling demonstrated reproducibility across all groups, with Jaccard indices ranging from 0.718 to 0.807, indicating stable cluster assignments. This four-group architecture was further evaluated by alternative hierarchical clustering, which yielded a moderate but substantive concordance with an adjusted Rand index of 0.524, confirming the underlying structural tendencies of the cohort despite algorithmic differences. As shown in **Table 1**, the overall cohort comprised predominantly middle-aged patients (median age: 47.25 years) with a median BMI of 26.96 kg/m^2^. The baseline demographic and clinical characteristics were comparable across the four clusters. There were no significant differences in age (*P* = 0.731), sex distribution (*P* = 0.437), or BMI (*P* = 0.936) among the groups. Furthermore, the prevalence of underlying comorbidities as well as lifestyle factors remained evenly distributed. Importantly, the county-level estimated residential altitude (*P* = 0.550), the categorized altitude groups (*P* = 0.893), and the presence of CMS clinical symptoms (*P* = 0.407) showed no statistical difference across the subsets.

**Table 1:** Baseline characteristics and comorbidities by clusters.

Characteristic	Overall (n = 198)	Cluster 1 (n = 20)	Cluster 2 (n = 62)	Cluster 3 (n = 84)	Cluster 4 (n = 32)	Method	*P*
Altitude, m	4316.04 (4294.93 - 4796.91)	4332.05 (4253.09 - 4840.61)	4321.63 (4269.51 - 4800.11)	4306.62 (4195.87 - 4767.50)	4324.04 (4306.62 - 4779.47)	Kruskal-Wallis	0.550
Age, years	47.25 (39.12 - 56.45)	46.45 (38.75 - 56.00)	47.00 (38.65 - 54.95)	48.25 (39.50 - 59.03)	47.40 (39.55 - 52.53)	Kruskal-Wallis	0.731
Gender, n (%)						Chi-square	0.437
Female	32 (16.2%)	2 (10.0%)	13 (21.0%)	14 (16.7%)	3 (9.4%)		
Male	166 (83.8%)	18 (90.0%)	49 (79.0%)	70 (83.3%)	29 (90.6%)		
BMI, kg/m^2^	26.96 (24.55 - 29.28)	26.96 (24.65 - 28.82)	26.74 (24.55 - 29.23)	26.96 (24.61 - 29.37)	26.96 (24.66 - 29.08)	Kruskal-Wallis	0.936
SBP, mmHg	131.25 ± 18.71	133.45 ± 25.45	132.10 ± 19.16	131.62 ± 17.39	127.25 ± 16.55	ANOVA	0.597
DBP, mmHg	87.00 (79.00 - 95.00)	89.50 (78.75 - 102.50)	87.50 (80.25 - 97.75)	87.00 (77.75 - 95.00)	84.50 (77.00 - 90.25)	Kruskal-Wallis	0.279
Altitude group, n (%)						Chi-square	0.893
<4500 (m)	129 (65.2%)	12 (60.0%)	40 (64.5%)	57 (67.9%)	20 (62.5%)		
≥4500 (m)	69 (34.8%)	8 (40.0%)	22 (35.5%)	27 (32.1%)	12 (37.5%)		
Ethnicity, n (%)						Chi-square	0.265
Other (including Han, Yi, etc.)	33 (16.7%)	2 (10.0%)	15 (24.2%)	11 (13.1%)	5 (15.6%)		
Tibetan	165 (83.3%)	18 (90.0%)	47 (75.8%)	73 (86.9%)	27 (84.4%)		
Smoking status, n (%)						Chi-square	0.657
Never	139 (70.2%)	16 (80.0%)	43 (69.4%)	56 (66.7%)	24 (75.0%)		
Former	21 (10.6%)	0 (0.0%)	6 (9.7%)	12 (14.3%)	3 (9.4%)		
Current	38 (19.2%)	4 (20.0%)	13 (21.0%)	16 (19.0%)	5 (15.6%)		
Current alcohol use, n (%)						Chi-square	0.130
No	147 (74.2%)	12 (60.0%)	42 (67.7%)	67 (79.8%)	26 (81.2%)		
Yes	51 (25.8%)	8 (40.0%)	20 (32.3%)	17 (20.2%)	6 (18.8%)		
Hypertension, n (%)						Chi-square	0.051
No	129 (65.2%)	16 (80.0%)	43 (69.4%)	46 (54.8%)	24 (75.0%)		
Yes	69 (34.8%)	4 (20.0%)	19 (30.6%)	38 (45.2%)	8 (25.0%)		
Regular use of antihypertensive medications, n (%)						Chi-square	0.075
No	165 (83.3%)	20 (100.0%)	50 (80.6%)	66 (78.6%)	29 (90.6%)		
Yes	33 (16.7%)	0 (0.0%)	12 (19.4%)	18 (21.4%)	3 (9.4%)		
Diabetes, n (%)						Chi-square	0.388
No	179 (90.4%)	18 (90.0%)	53 (85.5%)	79 (94.0%)	29 (90.6%)		
Yes	19 (9.6%)	2 (10.0%)	9 (14.5%)	5 (6.0%)	3 (9.4%)		
CMS, n (%)						Chi-square	0.407
No	125 (63.1%)	13 (65.0%)	40 (64.5%)	56 (66.7%)	16 (50.0%)		
Yes	73 (36.9%)	7 (35.0%)	22 (35.5%)	28 (33.3%)	16 (50.0%)		

Data are presented as mean ± standard deviation, median (interquartile range), or number (percentage) as appropriate. Normally distributed continuous variables were compared using one-way ANOVA; non-normally distributed variables were compared using the Kruskal-Wallis test; categorical variables were compared using the Chi-square test or Fisher’s exact test.

CMS, chronic mountain sickness; BMI, body mass index; SBP, systolic blood pressure; DBP, diastolic blood pressure.

### 3.2 Phenotypic characteristics, clinical symptoms and biochemical signatures

The biochemical markers and clinical symptom profiles of the four clusters are detailed in **Table 2**. The hemoglobin levels were elevated across all groups (overall median 219.00 g/L), with no significant inter-cluster difference observed (*P* = 0.164). Cluster 1 presented the highest absolute counts of neutrophils (9.88 ×10^9/L) and monocytes (0.76 ×10^9/L), accompanied by peak levels of systemic markers including CRP (117.70 mg/L) and D-dimer (2.02 mg/L), yet recorded a 0% incidence of breathlessness and/or palpitations.Cluster 2 exhibited the highest absolute lymphocyte count (1.85 ×10^9^/L), resulting in the lowest MLR (*P* < 0.001). Although presenting with relatively low systemic inflammatory markers (with the lowest D-dimer at 0.22 mg/L), this group recorded the highest incidence of breathlessness and/or palpitations (32.3%, *P* = 0.026). Cluster 3 presented as a relatively quiescent baseline phenotype, featuring the lowest absolute monocyte count (0.36 ×10^9/L), the lowest WBC count (5.17 ×10^9/L), and the lowest PLT count (120.00 ×10^9/L). Clinically, this group reported the lowest incidence of headache (10.7%). Cluster 4 demonstrated significant elevation in absolute eosinophils (0.23 ×10^9/L) and basophils (0.07 ×10^9/L), alongside the highest PLT levels (149.50 ×10^9/L) and the lowest CRP levels (5.97 mg/L). Clinically, this group exhibited the highest overall symptom burden, with a median CMS score of 2.50 and the highest incidence of headaches (31.2%).

**Table 2:** Phenotypic characteristics, clinical symptoms and biochemical signatures.

Characteristic	Overall (n = 198)	Cluster 1 (n = 20)	Cluster 2 (n = 62)	Cluster 3 (n = 84)	Cluster 4 (n = 32)	Method	*P*
Absolute neutrophil count, 10^^9^/L	3.63 (2.77 - 4.77)	9.88 (7.68 - 12.45)	3.29 (2.73 - 3.85) ^*^	3.60 (2.70 - 4.42) ^*^	3.63 (2.75 - 4.89) ^*^	Kruskal-Wallis	< 0.001
Absolute monocyte count, 10^9/L	0.45 (0.33 - 0.57)	0.76 (0.66 - 0.81)	0.41 (0.33 - 0.51) ^*^	0.36 (0.30 - 0.47) ^*^	0.56 (0.48 - 0.74) ^*,†,‡^	Kruskal-Wallis	< 0.001
Absolute lymphocyte count, 10^9/L	1.38 (1.06 - 1.75)	0.93 (0.79 - 1.23)	1.85 (1.58 - 2.04) ^*^	1.09 (0.91 - 1.32) ^†^	1.61 (1.32 - 2.05) ^*,‡^	Kruskal-Wallis	< 0.001
Absolute eosinophil count, 10^9/L	0.12 (0.06 - 0.20)	0.01 (0.01 - 0.05)	0.19 (0.12 - 0.24) ^*^	0.08 (0.04 - 0.11) ^*,†^	0.23 (0.17 - 0.30) ^*,‡^	Kruskal-Wallis	< 0.001
Absolute basophil count, 10^9/L	0.04 (0.02 - 0.05)	0.04 (0.03 - 0.05)	0.03 (0.02 - 0.04)	0.03 (0.02 - 0.04)	0.07 (0.06 - 0.09) ^*,†,‡^	Kruskal-Wallis	< 0.001
CMS Symptom Clinical Score	0.00 (0.00 - 3.00)	0.00 (0.00 - 3.00)	0.00 (0.00 - 3.75)	0.00 (0.00 - 3.00)	2.50 (0.00 - 3.00)	Kruskal-Wallis	0.283
headache, n (%)	35 (17.7%)	4 (20.0%)	12 (19.4%)	9 (10.7%)	10 (31.2%)	Chi-square	0.071
breathlessness and/or palpitations, n (%)	47 (23.7%)	0 (0.0%)	20 (32.3%) ^*^	18 (21.4%)	9 (28.1%)	Chi-square	0.026
sleep disturbance, n (%)	44 (22.2%)	3 (15.0%)	16 (25.8%)	17 (20.2%)	8 (25.0%)	Chi-square	0.706
paresthesia, n (%)	35 (17.7%)	2 (10.0%)	10 (16.1%)	17 (20.2%)	6 (18.8%)	Chi-square	0.725
tinnitus, n (%)	4 (2.0%)	0 (0.0%)	2 (3.2%)	1 (1.2%)	1 (3.1%)	Chi-square	0.716
Hb, g/L	219.00 (212.25 - 228.00)	219.50 (214.75 - 224.25)	217.00 (211.25 - 224.00)	220.00 (212.00 - 229.00)	226.00 (214.50 - 231.75)	Kruskal-Wallis	0.164
WBC, 10^9/L	5.75 (4.83 - 7.04)	11.82 (9.62 - 14.09)	5.75 (5.06 - 6.34) ^*^	5.17 (4.27 - 6.04) ^*,†^	6.34 (5.07 - 7.60) ^*,‡^	Kruskal-Wallis	< 0.001
PLT, 10^9/L	132.50 (107.25 - 158.25)	134.50 (104.25 - 164.25)	137.00 (111.50 - 161.50)	120.00 (97.75 - 148.75)	149.50 (118.25 - 168.50) ^‡^	Kruskal-Wallis	0.010
Neutrophil percentage, %	63.70 (56.12 - 71.50)	83.35 (79.80 - 87.72)	57.65 (53.40 - 61.10) ^*^	69.05 (61.98 - 73.75) ^*,†^	56.55 (53.10 - 64.85) ^*,‡^	Kruskal-Wallis	< 0.001
Lymphocyte percentage, %	24.70 (18.22 - 31.70)	8.45 (5.67 - 10.82)	31.85 (27.85 - 35.50) ^*^	21.55 (16.90 - 26.77) ^*,†^	28.00 (22.05 - 31.27) ^*,†,‡^	Kruskal-Wallis	< 0.001
Monocyte percentage, %	7.59 ± 2.42	6.54 ± 2.12	7.19 ± 1.86	7.35 ± 2.40	9.68 ± 2.57 ^*,†,‡^	ANOVA	< 0.001
Eosinophil percentage, %	2.15 (1.10 - 3.50)	0.10 (0.10 - 0.53)	2.90 (2.12 - 4.47) ^*^	1.50 (0.80 - 2.40) ^*,†^	3.55 (2.77 - 4.93) ^*,‡^	Kruskal-Wallis	< 0.001
Basophil percentage, %	0.60 (0.40 - 0.90)	0.30 (0.20 - 0.50)	0.50 (0.40 - 0.80) ^*^	0.60 (0.30 - 0.80) ^*^	1.05 (0.90 - 1.45) ^*,†,‡^	Kruskal-Wallis	< 0.001
NLR	2.55 (1.82 - 3.96)	9.96 (7.39 - 15.31)	1.85 (1.49 - 2.16) ^*^	3.22 (2.42 - 4.25) ^*,†^	1.95 (1.73 - 2.95) ^*,‡^	Kruskal-Wallis	< 0.001
MLR	0.31 (0.22 - 0.46)	0.81 (0.66 - 0.99)	0.22 (0.17 - 0.28) ^*^	0.35 (0.24 - 0.45) ^*,†^	0.34 (0.28 - 0.49) ^*,†^	Kruskal-Wallis	< 0.001
IBIL, μmol/L	15.50 (10.62 - 24.35)	22.40 (16.80 - 31.10)	14.70 (10.15 - 22.00) ^*^	17.10 (10.40 - 25.20)	13.45 (10.50 - 15.40) ^*^	Kruskal-Wallis	0.005
LDH, U/L	241.00 (198.00 - 316.00)	286.00 (207.75 - 466.00)	207.00 (187.00 - 264.00)	273.00 (219.50 - 334.50) ^†^	232.00 (186.00 - 291.00)	Kruskal-Wallis	0.001
D-dimer, mg/L	0.50 (0.24 - 1.48)	2.02 (1.53 - 3.37)	0.22 (0.17 - 0.35) ^*^	0.75 (0.41 - 1.79) ^†^	0.42 (0.30 - 0.67) ^*^	Kruskal-Wallis	< 0.001
CRP, mg/L	8.92 (4.46 - 30.65)	117.70 (95.00 - 178.50)	7.57 (4.69 - 9.30) ^*^	8.23 (4.18 - 24.50) ^*^	5.97 (4.72 - 21.05) ^*^	Kruskal-Wallis	0.005

*P < 0.05 compared with Cluster 1. ^†^*P* < 0.05 compared with Cluster 2. ^‡^*P* < 0.05 compared with Cluster 3.

Data are presented as mean ± standard deviation, median (interquartile range), or number (percentage) as appropriate. Normally distributed continuous variables were compared using one-way ANOVA; non-normally distributed variables were compared using the Kruskal-Wallis test; categorical variables were compared using the Chi-square test or Fisher’s exact test.

LDH, Lactate dehydrogenase; CRP, C-reactive protein; Hb, Hemoglobin; IBIL, Indirect bilirubin; PLT, Platelet count; WBC, White blood cell count; NLR, Neutrophil-to-lymphocyte ratio; MLR, Monocyte-to-lymphocyte ratio; CMS, chronic mountain sickness.

### 3.3 Correlation of clinical and biomarker phenotypes

Spearman correlation analysis was constructed to evaluate the relationships among environmental exposures, clinical symptoms, and biochemical markers (**Figure 2**). The analysis indicated that county-level estimated residential altitude did not show significant linear correlations with subjective symptom severity or with objective systemic inflammatory markers. Additionally, subjective neurological symptoms, such as sleep disturbance and paresthesia, were clustered independently from cardiopulmonary symptoms and coagulation indices in the correlation network.

**Figure 2:**
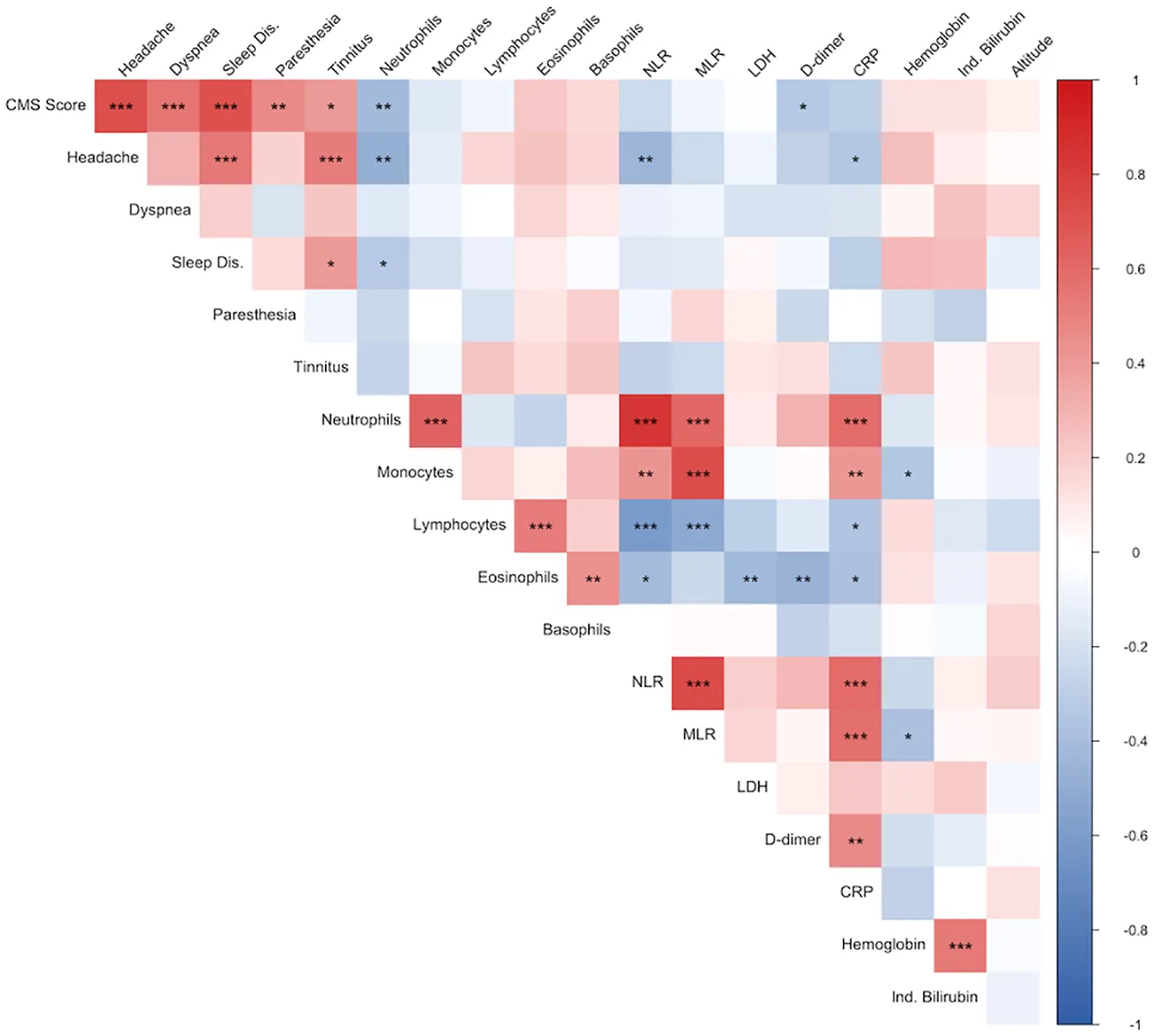
Spearman correlation network among environmental, clinical, and biochemical characteristics. LDH, Lactate dehydrogenase; CRP, C-reactive protein; NLR, Neutrophil-to-lymphocyte ratio; MLR, Monocyte-to-lymphocyte ratio; CMS, chronic mountain sickness. *P < 0.05, **P < 0.01, ***P < 0.001.

### 3.4 Independent predictors of highly symptomatic cardiopulmonary phenotype

Given the high risk of severe cardiopulmonary symptoms associated with Cluster 2, we evaluate the independent predictive capacity of MLR for identifying this specific phenotype. The median MLR of the cohort was 0.311, with an interquartile range of 0.223 to 0.455. We utilized a pre-specific Firth penalized multivariable logistic regression model, categorizing MLR into quartiles, to address limited outcome events (**Table 3**). In the fully adjusted model incorporating clinically relevant covariates including age, BMI, residential altitude, baseline Hb, smoking status, and drinking status, patients into the lowest quartile (Q1) exhibited a significantly elevated risk of belonging to the highly symptomatic phenotype compared to those in the highest quartile (Q4, reference), with an odds ratio (OR) of 33.764 (95% CI: 9.615-180.993, P<0.001) (**Figure 3**). A clear dose-response gradient was observed across quartiles. Neither absolute altitude (*P* = 0.431) nor hemoglobin level (*P* = 0.072) emerged as significant independent predictors. The prediction model demonstrated excellent calibration via the Hosmer-Lemeshow test (X-squared=5.119, *P* = 0.745). Initial ROC curve analysis revealed an AUC of 0.829 for MLR, which remained robust at 0.810 following internal validation via 1000 bootstrap resamples (**Figure 4**).

**Table 3:** Independent predictors of the highly symptomatic cardiopulmonary phenotype (Cluster 2) utilizing Firth penalized logistic regression.

Variable	Firth model OR (95% CI)	*P*-value
MLR quartiles		
MLR Quartile 4 (Highest)	1.000 (Reference)	–
MLR Quartile 3	4.217 (1.068 - 23.549)	0.039
MLR Quartile 2	12.154 (3.503 - 64.119)	< 0.001
MLR Quartile 1 (Lowest)	33.765 (9.615 - 180.995)	< 0.001
Covariates		
Age (per 10 years increase)	0.868 (0.626 - 1.187)	0.379
Body Mass Index (kg/m^2^)	0.976 (0.911 - 1.041)	0.476
Residential Altitude (per 100m increase)	0.973 (0.908 - 1.042)	0.431
Hemoglobin (g/L)	0.977 (0.952 - 1.002)	0.072
Smoking Status (Former) ^*^	0.587 (0.158 - 2.003)	0.399
Smoking Status (Current)^*^	0.566 (0.188 - 1.622)	0.293
Current alcohol use (Yes) ^†^	2.202 (0.884 - 5.613)	0.090

The highest MLR quartile (Q4), non-smoking status, and non-drinking status served as the reference categories.

^*^ Smoking categories (Former and Current) represent the categorical transformation of smoking status compared to non-smokers.

^†^ Current alcohol use represents active alcohol consumption compared to non-drinkers.

MLR, Monocyte-to-lymphocyte ratio; OR, odds ratio; CI, confidence interval.

**Figure 3:**
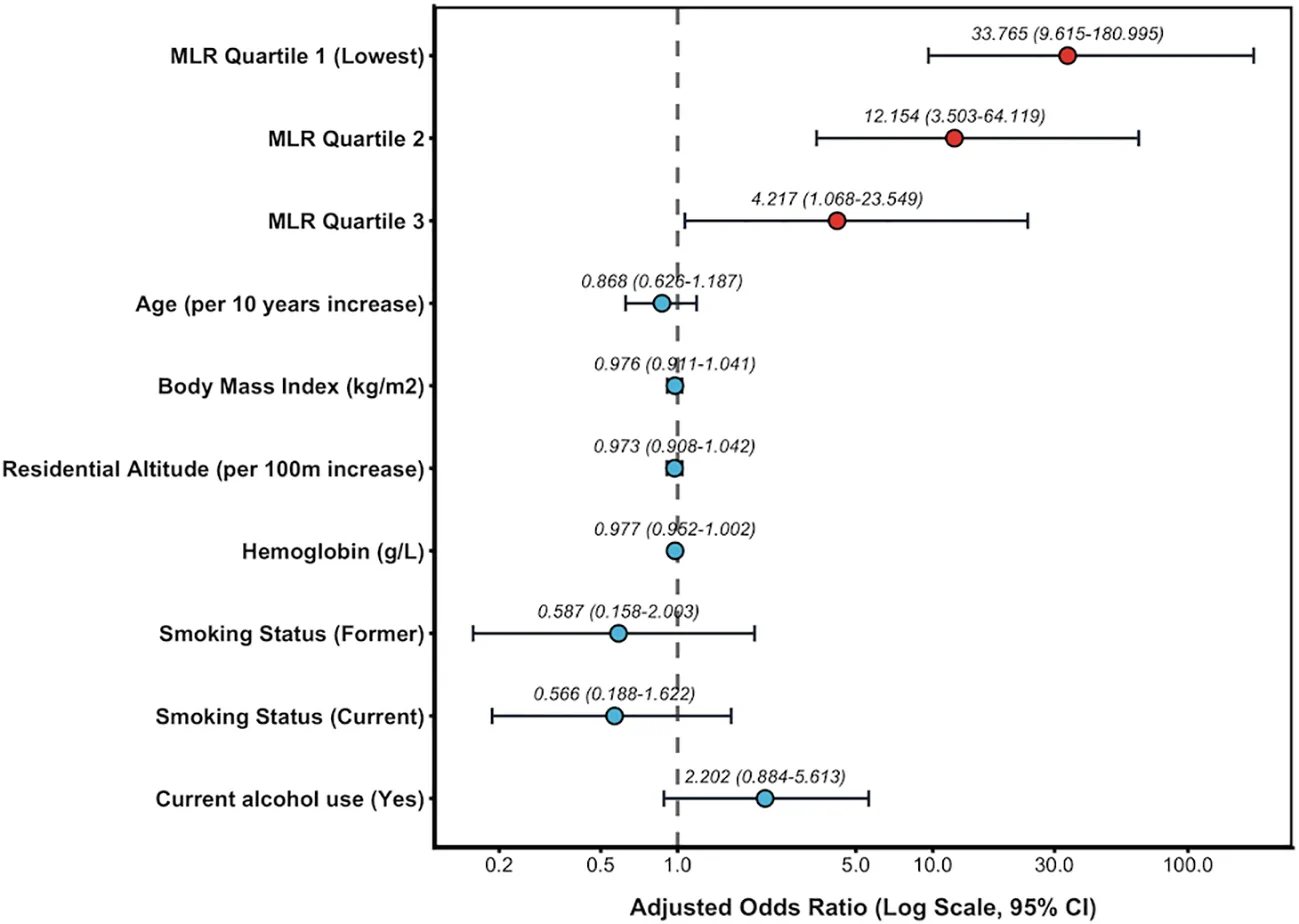
Forest plot illustrating the independent predictors of the highly symptomatic cardiopulmonary phenotype (Cluster 2). The fully adjusted model incorporates MLR quartiles, age (per 10 years increase), BMI, residential altitude (per 100m increase), baseline hemoglobin, smoking status (former/current), and current alcohol use. The highest MLR quartile (Q4), non-smoking, and non-drinking statuses served as the reference categories. The adjusted OR and 95% CI are plotted on a logarithmic scale. To ensure robust clinical interpretability and consistent scaling, continuous variables were scaled as follows: monocyte-to-lymphocyte ratio (MLR, per quartile), age (per 10-year increase), and residential altitude (per 100-meter increase). The horizontal error bars represent the 95% CI. Red highlighting indicates a significant independent association (*P* < 0.05). MLR, monocyte-to-lymphocyte ratio; BMI, body mass index; OR, odds ratios; CI, confidence intervals.

**Figure 4:**
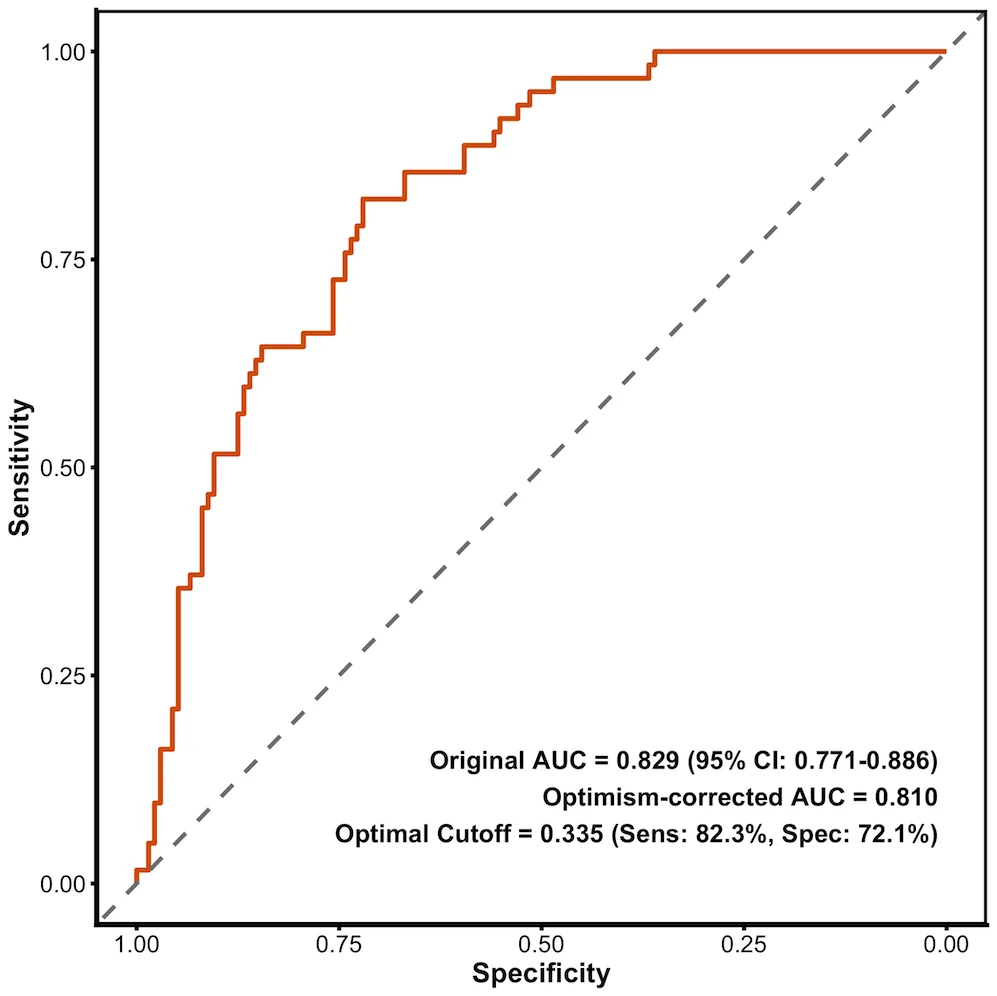
Diagnostic performance of MLR for identifying the highly symptomatic cardiopulmonary phenotype (Cluster 2). The ROC curve analysis demonstrated an initial AUC of 0.829. Following robust internal validation utilizing 1,000 bootstrap resamples, the optimism-corrected AUC remained high at 0.810, indicating excellent model stability and diagnostic discrimination. The optimal cutoff value (determined by the maximum Youden Index) along with its corresponding sensitivity and specificity are detailed within the figure. MLR, monocyte-to-lymphocyte ratio; AUC, area under the curve; ROC, receiver operating characteristic.

## 4 Discussion

In the present study, we employed an unsupervised machine learning approach based on peripheral leukocyte subsets to stratify patients with HAPC into four distinct immunophenotypes. We further identify a unique, highly symptomatic cardiopulmonary phenotype, Cluster 2, and demonstrated that the MLR serves as a potential independent predictor for this specific subtype. These findings offer a novel immunologic perspective on the heterogeneity of chronic altitude illness and provide an easily accessible biomarker for early clinical triage.

It is interesting to notice that, despite all enrolled patients meeting the established diagnostic criteria for HAPC, they exhibited substantial heterogeneity in clinical symptoms and biochemical inflammatory profiles. Traditionally, the severity of CMS and associated organ dysfunction is assumed to correlate linearly with the magnitude of hypoxemia and the degree of excessive erythropoiesis (Villafuerte and Corante, 2016). Under this traditional paradigm, one might expect a tight mechanistic coupling between extreme Hb elevation and pronounced systemic inflammation driven by hyperviscosity-induced microcirculatory shear stress and subsequent endothelial activation. However, our findings reveal a phenotypic decoupling. Despite all patients presenting with extreme polycythemia (overall median Hb 219.00 g/L), Hb levels did not significantly differ across the four distinct immunophenotypic clusters (*P* = 0.164), nor did Hb emerge as an independent predictor in the multivariable Firth regression model. However, our finding reveals a phenotypic decoupling phenomenon. This suggests that once the threshold of chronic altitude maladaptation is crossed, the specific trajectory of end-organ complications is driven by intrinsic host immune remodeling rather than a simple dose-response to the absolute hypoxic or erythropoietic burden (Hsieh et al., 2016; Wang et al., 2026). In the present cohort, neither hemoglobin level nor county-level estimated residential altitude differentiated the identified immunophenotypes (Gonzales, 2025; Zhou et al., 2025; Zubieta-Calleja, 2025), highlighting the potential value of immune-based indices such as MLR for identifying clinically distinct HAPC subgroups. However, given the limited spatial resolution of our altitude measurement, these findings should not be interpreted as evidence that individual altitude exposure is unrelated to clinical heterogeneity.

The diversity of these immune trajectories is reflected in the distinct pathophysiological mechanisms underlying the four clusters. Cluster 1 represents a classical “systemic hyper-inflammatory” baseline, exhibiting the highest absolute counts of neutrophils and monocytes, along with peak levels of CRP and D-dimer. However, these patients reported the lowest incidence of breathlessness, suggesting that while the systemic inflammatory cascade is highly activated, it may remain confined to the intravascular space without causing massive pulmonary endothelial breach (Gonzalez and Wood, 2010; Brewster et al., 2023). The intact endothelial barrier prevents the extensive infiltration of inflammatory cells into end-organs, thereby preserving pulmonary microcirculation and avoiding symptomatic breathlessness (Richalet et al., 2023; Karki and Birukova, 2018). Cluster 3, constituting the largest proportion of our cohort, appears to present the “stable compensated” HAPC phenotype. Characterized by the lowest absolute counts in several leukocyte lineages and modest systemic inflammatory markers, this group exhibits a moderate but mixed symptom burden. This reflects the typical baseline state where extreme erythrocytosis is tolerated without triggering severe immune polarization (Facco et al., 2005; Yi et al., 2021; Feng et al., 2023). Interestingly, Cluster 4 emerges as an “allergic-like” state, notable for significantly elevated eosinophil and basophil counts, along with the highest overall CMS scores and headache incidence. Chronic intermittent hypoxia is known to induce eosinophil activation and degranulation (Nissim Ben Efraim et al., 2010, 2013), which can mediate vascular leakage and exacerbate generalized systemic symptom burden without necessarily driving traditional inflammatory markers like CRP (Koike et al., 2022).

In contrast, Cluster 2 embodies a distinct and highly symptomatic phenotype. Interestingly, these patients do not possess the absolute lowest counts of circulating monocytes, rather exhibit a proportional shift resulting in the lowest MLR across all groups. Despite having quiescent systemic inflammatory markers, these patients suffer from the highest incidence of cardiopulmonary symptoms, performing as a 32.3% incidence of severe breathlessness and palpitations. We hypothesize that the abnormally low MLR in this group is not a reflection of true immune depletion, but rather an illusion caused by massive margination and active transendothelial migration of circulating monocytes into the pulmonary microvasculature (Kreisel et al., 2010; Systemic hypoxia drives inflammation persistence via suppression of monocytes, 2022). However, this concept of target-organ sequestration remains an untested hypothesis in the context of HAPC and requires objective validation. If this mechanism occurs similar to well documented processes in acute lung injury, this localized pulmonary sequestration and subsequent macrophage differentiation could potentially trigger microvascular remodeling, ventilation-perfusion mismatch, and right ventricular strain (Jobse et al., 2014; Florentin et al., 2018; Chen et al., 2024; Tan et al., 2026), ultimately leading to severe localized cardiopulmonary symptoms while leaving the peripheral blood appearing unremarkable, which is the well-documented mechanism in acute lung injury (O’Dea et al., 2009; Wilson et al., 2009; Grommes and Soehnlein, 2011).

The translation of these pathophysiological insights into clinical practice relies heavily on the predictive value of MLR, particularly for identifying the highly symptomatic Cluster 2. Extensive previous research has established MLR as an established prognostic indicator for systemic inflammation, endothelial dysfunction, and cardiovascular diseases such as coronary artery disease (Chen et al., 2019; Bani Hani et al., 2025; Kalhor et al., 2025). Furthermore, emerging evidence has underscored the clinical utility of MLR in critical respiratory conditions, demonstrating its association with mortality in critically ill patients suffering from acute respiratory failure (Xiao et al., 2026). Despite the ongoing scientific discourse regarding the precise role of inflammatory cascades in the pathogenesis of severe high-altitude illnesses (Swenson et al., 2002), to the best of our knowledge, MLR has never been applied to the risk stratification of HAPC or CMS. In the resource-limited primary care setting of high-altitude regions, advanced diagnostic modalities like right heart catheterization or multiple cytokine profiling are largely inaccessible. MLR provides a practical alternative to this barrier, as it can be effortlessly and inexpensively derived from a routine complete blood count. Our pre-specified Firth penalized multivariable analysis confirmed that MLR retains its predictive power independently of age, BMI, altitude, and hemoglobin levels, making it a readily accessible tool for grassroots clinical screening.

By applying unsupervised clustering to an independent HAPC cohort expanded by one year, we address the immunophenotypic heterogeneity left unanswered by our prior prediction based on red cell distribution width (Wu et al., 2026). While our previous work established a single hematological parameter (RDW) as a general screening tool, the current study fundamentally advances this paradigm by moving beyond single-marker risk assessment. Here, we systematically deconstruct the inflammatory heterogeneity of HAPC into four distinct immunophenotypes, thereby identifying a highly specific, symptom-burdened cardiopulmonary subpopulation. Within this novel phenotypic framework, MLR serves as a highly actionable biomarker for a hidden symptomatic cardiopulmonary subgroup.

Nevertheless, several limitations must be acknowledged. First, the cross-sectional, observational design precludes inferences between MLR, leukocyte sequestration, and severe cardiopulmonary symptoms. Additionally, because statistical corrections for multiple testing were not applied, the between-group biomarker comparisons should be interpreted as exploratory. Second, our definition of the severe cardiopulmonary phenotype relied exclusively on subjective symptom reporting. Due to retrospective data limitations, the modified 5-item symptom score lacks formal concordance validation with the standard Qinghai CMS score, restricting direct comparisons with other studies. Consequently, these symptoms cannot be directly equated with structural end-organ damage, and the “target-organ sequestration” hypothesis requires future histopathological or hemodynamic validation. Thirdly, the relatively small sample size per cluster and the data-driven, exploratory nature of unsupervised machine learning mean these four immunophenotypes represent a conceptual framework rather than a universally established classification. Fourth, the 11-year study period introduces potential temporal biases; although Z-score transformations mitigated instrumental batch effects, the lack of granular temporal data precluded a formal time-trend analysis. Furthermore, utilizing county-level average altitude as a proxy for individual exposure may introduce measurement errors. Finally, the strict exclusion of patients with pre-existing cardiopulmonary comorbidities, while necessary to isolate HAPC-specific immunophenotypes, limits the generalizability of our findings to broader, real-world populations. Future multi-center, prospective longitudinal cohorts incorporating objective cardiopulmonary testing, *in vivo* imaging, or tissue biopsies are warranted to further elucidate the precise mechanistic pathways driving these distinct immunophenotypes.

## 5 Conclusion

High altitude polycythemia is not a homogeneous disease, but rather a syndrome characterized by significant clinical and pathophysiological heterogeneity. This intrinsic heterogeneity manifests across diverse trajectories of systemic inflammatory responses and subjective symptom burdens, independent of the absolute magnitude of erythrocytosis or environmental hypoxia. MLR serves as a potential independent predictor for identifying the most severe symptomatic cardiopulmonary phenotype, holds significant potential as a novel, highly accessible clinical index for the risk stratification and prevision evaluation of CMS in high-altitude medicine.

## Data Availability

The raw data supporting the conclusions of this article will be made available by the authors, without undue reservation.
